# Evaluation of NDT Methods for In Situ Documentation of Concrete for Reuse: Laboratory Studies

**DOI:** 10.3390/ma18112470

**Published:** 2025-05-24

**Authors:** Serkan Karatosun, Thomas Ingeman-Nielsen, Lisbeth M. Ottosen

**Affiliations:** Department of Environment and Resource Technology, DTU Sustain, Technical University of Denmark, Brovej, Building 118, 2800 Lyngby, Denmark; thin@dtu.dk (T.I.-N.); limo@dtu.dk (L.M.O.)

**Keywords:** concrete, reuse, NDT, ultrasonic pulse velocity, rebound hammer, electrical resistivity

## Abstract

Concrete production has significant environmental impacts due to extensive raw material use and high CO_2_ emissions. Reusing structural concrete elements can potentially reduce these environmental impacts by reducing the demand for new production. However, reliable and practical documentation of concrete properties is needed for safe and scalable reuse. Although several non-destructive testing (NDT) methods show promise for in situ assessment of concrete properties, a clear gap remains in implementing them into a comprehensive approach for reuse documentation. This study investigates the potential of combining ultrasonic pulse velocity (UPV), rebound hammer (RH), and electrical resistivity (ER) methods for documenting concrete properties for reuse. Several parameters relevant to reuse scenarios, such as saturation level and aggregate type and size, were systematically evaluated to understand their impact on NDT documentation of concrete for reuse. NDT documentation of compressive strength and chloride migration coefficient was assessed on 120 cylindrical specimens. Fifteen concrete mixtures were used with three aggregate compositions and five water–cement ratios. The experimental results are discussed in the context of in situ documentation of structural elements in donor buildings to ensure the practical applicability of the findings. The findings show that these NDT methods can potentially document the properties of concrete reliably and practically, thereby addressing the lack of in situ documentation procedures needed to enable the safe and scalable reuse of structural elements.

## 1. Introduction

Concrete production has significant environmental impacts, requiring large amounts of raw materials and energy and causing substantial CO_2_ emissions during cement production [1,2]. Recent reports show that the buildings and construction sector account for 21% of global greenhouse gas emissions, with cement and other concrete elements responsible for over half of this climate footprint [3,4]. Despite its heavy environmental impact, concrete remains one of the most widely used construction materials globally, with an estimated 30 billion tons of consumption annually [5]. Due to its favorable material properties and as a cheap, locally available resource, concrete will continue to be widely used [6]. In some parts of the world, most reinforced concrete wastes are recovered by downcycling, mainly by crushing the concrete, using it as aggregate, and treating the reinforcement as scrap metal [7,8,9,10,11]. Reusing the existing concrete structural elements, such as beams, columns, and walls, has emerged as an environmentally and economically better alternative to downcycling [7,10,12,13]. Reusing existing structural elements in new constructions can potentially reduce waste from demolitions and, most notably, minimize the environmental impact of producing new concrete [12,14].

A critical challenge in implementing structural concrete reuse is the need for reliable and practical documentation of concrete properties [15,16,17]. Documentation should comprehensively cover concrete mechanical properties, reinforcement layout, visible damage or defects, and durability-related factors such as carbonation and chloride ingress to enable safe reuse [18]. The conventional approach to evaluating mechanical properties is core sampling and conducting laboratory tests on these core samples. However, core sampling evaluates a single point in a concrete element and is not optimal in a reuse context, as documenting the uniformity of the whole concrete element for reuse would require extensive sampling. Moreover, core extraction is a destructive method that may compromise the structural integrity of elements and reduce their reuse potential. Therefore, relying on core sampling is neither practical nor economically feasible, and presents a barrier to the safe and scalable reuse of structural elements.

Non-destructive testing (NDT) presents a practical solution to the challenge of documenting the properties of structural concrete elements for reuse [18]. NDT methods have been widely used in civil engineering to assess various properties of structural concrete elements [19,20,21]. Ultrasonic pulse velocity (UPV), rebound hammer (RH), and electrical resistivity (ER) showed strong potential for NDT reuse documentation [18]. UPV and RH methods were commonly conducted to estimate compressive strength [22], while ER was used to evaluate durability characteristics, such as resistance to chloride ingress [23].

Several studies have shown that UPV effectively detected internal voids and assessed homogeneity but tended to underestimate compressive strength due to sensitivity to internal defects [24,25]. Conversely, RH often overestimated strength due to surface carbonation [25], though this could be mitigated by using correction factors [26,27]. Combining UPV and RH in the SonReb method improved accuracy by compensating for individual limitations, particularly when aggregate and moisture conditions were unknown [28,29].

Electrical resistivity, particularly surface resistivity, has also gained recognition for estimating chloride ingress risk [23,30]. However, its accuracy in field conditions was influenced by moisture variability and internal inconsistencies within elements [30,31,32], which can limit its reliability in reuse documentation. These limitations are especially critical when comparing elements with unknown exposure histories or moisture gradients, as often encountered in reuse scenarios.

Previous studies have mainly focused on evaluating the overall condition of entire structures rather than documenting the material properties of individual structural elements intended for reuse. The existing literature, therefore, lacks a reuse documentation methodology that enables reliable assessment at the element scale. Furthermore, a clear gap remains in combining multiple NDT methods into a comprehensive and practical approach designed explicitly for reuse documentation [18].

Moreover, several concrete characteristics, e.g., water saturation (from here on referred to as saturation level) and aggregate type and size, strongly influenced the NDT results and their relationship with target properties [19,31]. These properties usually vary between buildings and even within the same structure, presenting challenges in reuse where material properties might be unknown. Therefore, systematic research into how these parameters influence NDT measurements is necessary to develop reliable documentation procedures for reuse.

This laboratory study aims to evaluate the use of the NDT methods UPV, RH, and ER for in situ documentation of structural concrete elements intended for reuse. Compressive strength and chloride migration coefficient are important properties when establishing documentation for reuse. The Sonic-Rebound method (SonReb), combining UPV and RH, was used to estimate compressive strength. ER measurements were used to estimate the chloride migration coefficient. Additionally, the effects of saturation level and aggregate type and size on the results of these NDT methods and their relationships with target properties were evaluated.

This study addresses the lack of documentation methods for reusing structural concrete elements by systematically investigating parameters associated with reuse cases. The findings highlight the efficiency of using NDT methods for in situ documentation of structural concrete elements for reuse. The novelty is that the experimental results are thoroughly discussed, focusing on in situ documentation of structural elements in the donor building, ensuring the findings enable safe and scalable structural element reuse.

## 2. Materials and Methods

### 2.1. Casting of Test Specimens

Fifteen concrete mixtures were prepared to systematically investigate how reuse-related parameters influence the relationships between target properties and NDT measurements. The mixtures included five different water-to-cement (*w*/*c*) ratios and three types of aggregate compositions, as detailed in Table 1. These mixture designs reflect conditions commonly encountered in reuse projects and account for the variability observed during in situ evaluations. This approach aims to ensure that the findings can effectively inform the development of practical reuse documentation procedures.

**Figure 1:**
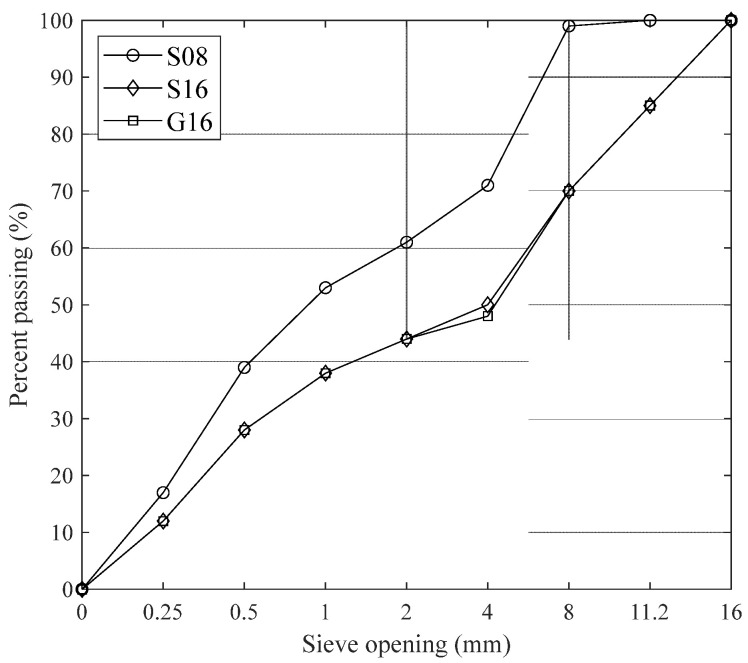
Gradation curves of three aggregate compositions: S08 (○), S16 (◊), and G16 (□).

Ten cylinders with a diameter of 10 cm and a length of 20 cm were cast with each mixture, and each set of ten cylinders is referred to as a batch from here on. Crushed granite (5 batches) or sea-dredged gravel (sea stone, 10 batches) were used as coarse aggregates since they are the most often-used aggregate types in the region (Denmark) [33]. The maximum aggregate sizes, D_max_, were 8 mm (5 batches) or 16 mm (10 batches). The aggregate gradation curves are presented in Figure 1.

To ensure comparable consistency across all mixtures, varying dosages of a polycarboxylate ether (PCE)-based superplasticizer (Master Glenium SKY 851, Master Builders Solutions, Rodekro, Denmark) were added. After 28 days of water curing at 20 ± 2 °C, all specimens were stored and tested in the same controlled laboratory environment (20 ± 2 °C, 35–55% relative humidity). The specific ages of the specimens during testing are reported in the methodology section.

### 2.2. NDT Methods

The ultrasonic pulse velocity (UPV) test involves sending an ultrasonic wave through a concrete specimen and measuring the travel time between the two transducers [34]. The velocity depends on the density and elastic properties of the concrete, providing information on compressive strength and elastic modulus [35]. UPV measurements were conducted following EN 12504-4:2021 [36]. PUNDIT Lab equipment (Proceq, Zurich, Switzerland) was used, while a water-based Dane-Gel R2 coupling gel was used to achieve good contact with transducers and cylinders. Round transducers (54 kHz) were used. Transmission time was measured between two opposite ends of the cylinders, i.e., direct transmission. The average of the five readings was taken as the mean ultrasonic pulse velocity.

The rebound hammer (RH) method involves striking a concrete surface with a spring-loaded hammer and measuring the rebound value [34]. The rebound value indicates surface hardness; higher values typically correspond to harder concrete and higher compressive strength [37]. RH measurements were conducted following EN 12504-2:2021 [38] using a Silver Schmidt hammer (Proceq, Zurich, Switzerland), which provides a rebound number Q. The median of ten readings was reported as the representative rebound value. The samples were placed in a compression machine during testing to provide rigid support and minimize movement or vibration, which could otherwise affect the rebound readings. To evaluate the effect of saturation level, multiple measurements were performed on each sample at different stages of the experiment. Since each strike leaves an indentation mark and alters the surface condition, all measurements were taken on previously untested areas of the sample surface.

The electrical resistivity (ER) method measures the electrical resistance of concrete by applying an electric current and measuring the resulting voltage drop [34]. Resistivity is then calculated using this resistance and a factor that depends on the geometry of the sample and test setup [39]. The ER of concrete is influenced by its pore structure, chemical composition, and moisture content [23]. It is beneficial for assessing durability-related properties, such as resistance to chloride ingress [23,40], which are essential for evaluating the reuse potential of concrete elements.

ER measurements were performed using a Resipod (Proceq, Zurich, Switzerland) in surface and bulk resistivity configurations to enable comparison of the results. The device operates with a 40 Hz alternating current and a maximum voltage of 38 V. Surface resistivity was measured using the Wenner four-probe method, with two different inter-probe spacings (a = 38 mm and a = 50 mm) to assess measurement consistency. The inter-probe spacing was adjusted using the Resipod Geometric (Proceq, Zurich, Switzerland), which allows for customizable probe spacing [41]. In this setup, current is injected through the outer electrodes while the inner electrodes record the voltage drop. Forty readings were performed per specimen (ten each at the 0°, 90°, 180°, and 270° positions around the cylinder), and their mean was reported as the representative surface resistivity. For bulk resistivity measurements, the two-plate method was applied. The cylindrical specimen was positioned between two steel plates, which functioned as both current injection and potential electrodes. The average of ten readings was reported as the representative bulk resistivity.

### 2.3. Test Methodology

**Figure 2:**
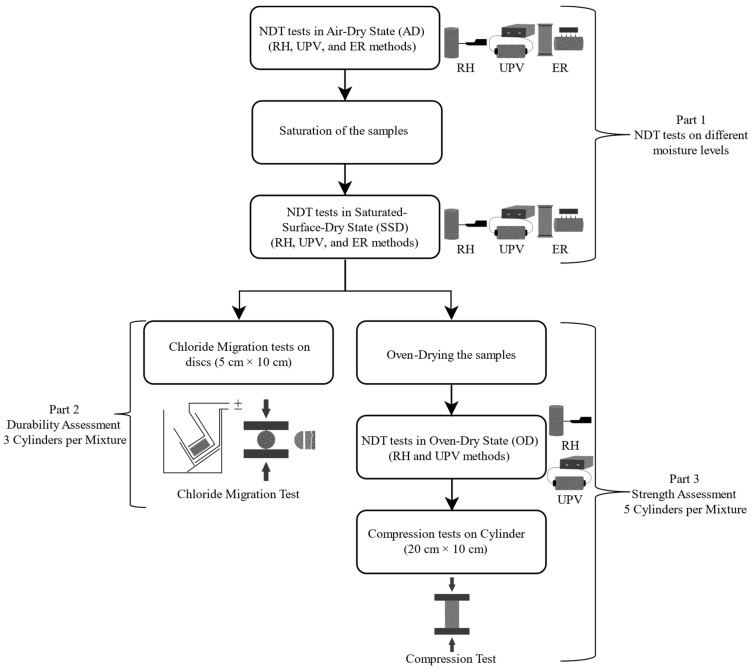
Flow chart of the experimental program.

The experimental program consisted of three parts. Figure 2 shows the workflow of the experimental procedure. In the first part, all three NDT methods were applied to specimens at different saturation levels. The second and third parts focused on obtaining the chloride migration coefficient and compressive strength values, respectively.

In the first part, RH, UPV, and ER measurements were carried out on the air-dry (AD) specimens. The specimens were then saturated by submersion in water for three days. After saturation, excess surface water was removed, and the specimens were brought to a saturated-surface-dry (SSD) condition. The NDT measurements were repeated on the specimens in the SSD state to investigate the effect of saturation.

**Figure 3:**
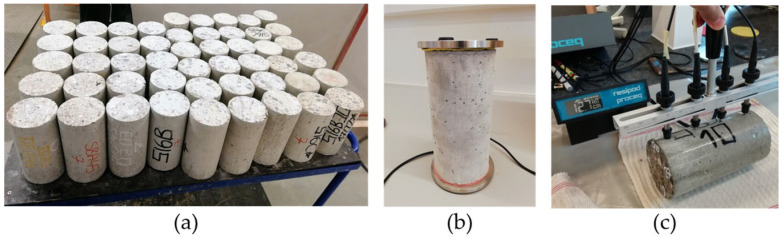
NDT of durability assessment. (**a**) Samples used for part 2, (**b**) Bulk resistivity test, (**c**) Surface resistivity test with adjustable electrode spacing.

**Figure 4:**
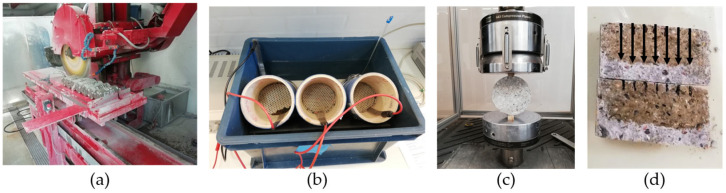
Chloride migration test of durability assessment: (**a**) Sawing 5 cm discs, (**b**) NT Build 492 Setup, (**c**) Splitting samples after NT Build 492 test, (**d**) Cross-section of a concrete specimen after splitting and spraying with silver nitrate solution to visualize chloride ingress. Arrows show chloride ingress direction from the exposed surface.

In the second part, three cylindrical specimens of each mixture were used to assess the non-steady state chloride migration coefficient (D_nssm_). Experiments in this part were conducted when the samples were at the age of 120 days. A concrete disc, five cm long, was cut from the middle of each specimen after finishing part 1. The D_nssm_ values of these discs were determined according to NT Build 492 [42]. This method involves applying a voltage to migrate chloride ions through the concrete disc. Then, disc samples are removed and split. The penetration depth of chloride ions is measured by spraying silver nitrate solution on freshly split surfaces. D_nssm_ is calculated using the penetration depth, duration of the procedure, voltage level, and temperature change in the solution. Figure 3 and Figure 4 show the test for durability assessment.

In the trial tests, it was observed that the duration suggested by NT Build 492 [42] (24 h) resulted in full penetration of chloride ions throughout all specimens. These results were not appropriate for the assessment. Since the samples have high permeability due to high w/c ratios (reaching up to 0.84), and considering the trial test results, the chloride migration duration was set to 6 h for all samples. This adjustment aimed to align the testing conditions and prevent excessive chloride penetration.

**Figure 5:**
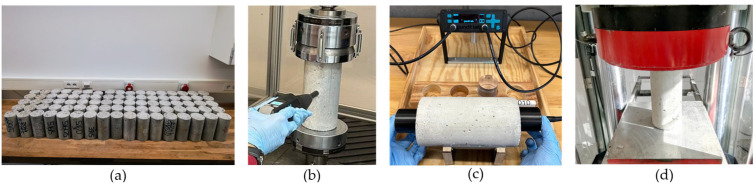
Compressive strength assessment part: (**a**) Samples used for part 3, (**b**) RH test, (**c**) UPV test, (**d**) Compression test.

The third part was conducted using five cylindrical specimens of each type. Experiments in this part were conducted when the samples were at the age of 300 days. The specimens were placed in ovens to dry (105 °C). The specimens were left in the ovens until they reached a constant weight, showing they were in the oven-dry (OD) state. UPV and RH tests were conducted on specimens in the oven-dry state. However, the ER measurements on the OD state were not stable due to the lack of moisture in the specimens. Finally, compression testing was conducted on the specimens to obtain the actual compressive strength values (Figure 5). Compression tests were performed using a compression machine (Toni Technik, Berlin, Germany) with a maximum loading capacity of 3 MN. The loading rate was 0.6 MPa/s.

### 2.4. Statistical Data Analysis

Univariate and multivariate regression analyses were performed using linear or power functions. The adjusted coefficient of determination ($R_{adj}^{2}$) and root mean square error (RMSE) were used to evaluate the goodness of fit. $R^{2}$ measures the proportion of variance in the dependent variable that is predictable from the independent variable. However, R^2^ tends to increase with the addition of more predictors, regardless of whether they contribute meaningfully to the model. Therefore, the adjusted coefficient of determination ($R_{adj}^{2}$) was used, as it accounts for both the number of predictors and the sample size, providing a more reliable evaluation of model performance. $R^{2}$, $R_{adj}^{2}$, and RMSE were calculated using Equations (1)–(3).

(1) $$R^{2}=1-\left(\frac{\sum_{i=1}^{n}{\left(y_{i}-{\hat{y}}_{i}\right)}^{2}}{\sum_{i=1}^{n}{\left(y_{i}-\bar{y\;}\right)}^{2}}\right)$$

(2) $$R_{adj}^{2}=1-\frac{n-1}{n-p}(1-R^{2})$$

(3) $$RMSE=\sqrt[{}]{\frac{{\left(y_{i}-{\hat{y}}_{i}\right)}^{2}}{n-p}}$$

where $y_{i}$ is the measured data, ${\hat{y}}_{i}$ is the value predicted by the model, $\bar{y}$ is the mean value of the measured data, *n* is the number of data points, and *p* is the number of independent variables in the model.

## 3. Results

Table 2 presents the descriptive statistics of the NDT measurements, compressive strength, and chloride migration coefficients. The table includes minimum, maximum, mean, median, and standard deviation values for each parameter. UPV, RH, and compressive strength measurements were obtained using five cylinders per mixture. ER and chloride migration measurements were obtained using three cylinders per mixture. The following sections present the correlations between NDT measurements and the corresponding target properties: compressive strength with UPV and RH, and chloride migration coefficient with ER. These measurements were obtained from the same specimens at the same age to ensure consistency and eliminate age-related discrepancies.

The UPV and RH results are reported under three different saturation levels to capture the influence of moisture content. ER measurements include surface resistivity at two electrode spacings ($\rho_{50}$ and $\rho_{38}$) and bulk resistivity ($\rho_{b}$), all conducted in the saturated surface dry (SSD) condition. Measurements in the oven-dry (OD) and air-dry (AD) states were excluded due to instability.

### 3.1. Correlation of Compressive Strength with UPV and RH

**Figure 6:**
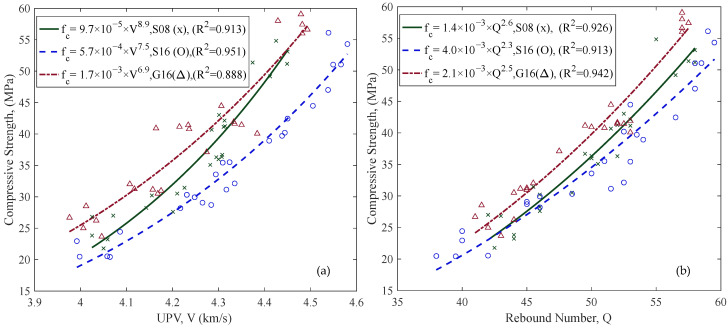
Relationships between compressive strength and NDT results in the AD state: (**a**) UPV, (**b**) RH, for different aggregate compositions (green = S08, blue = S16, red = G16).

Correlations between compressive strength and UPV and RH results in the AD state for different aggregate compositions are shown in Figure 6. Power law regression models were fitted to each aggregate group (S08, S16, G16), showing strong correlations in all cases (R^2^ values ranging from 0.888 to 0.951). The highest R^2^ value was observed for S16 with UPV (R^2^ = 0.951) and for G16 with RH (R^2^ = 0.942).

**Figure 7:**
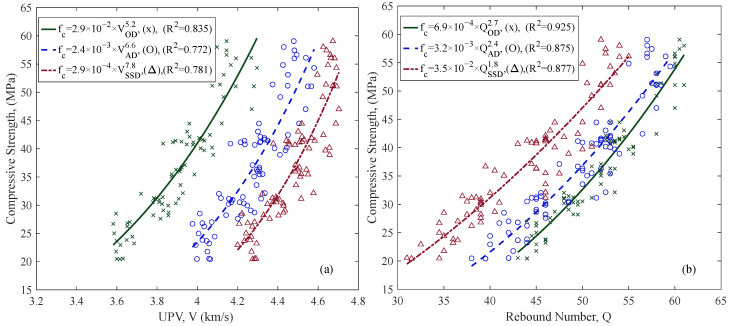
Relationships between compressive strength and (**a**) UPV, and (**b**) RH, for specimens in different saturation levels (green = OD, blue = AD, red = SSD). Data are shown with all aggregate groups combined.

UPV and RH tests were conducted at three different saturation levels: oven-dried (OD), air-dried (AD), and saturated-surface-dry (SSD) (see Figure 2). Figure 7 shows the notable effect of saturation levels on the relationships of compressive strength with UPV and RH values. However, the effects were in the opposite direction for the two methods. While UPV results increased with saturation, RH results decreased. Measurements on the OD specimens exhibited stronger correlations than the other saturation levels for both UPV and RH.

**Figure 8:**
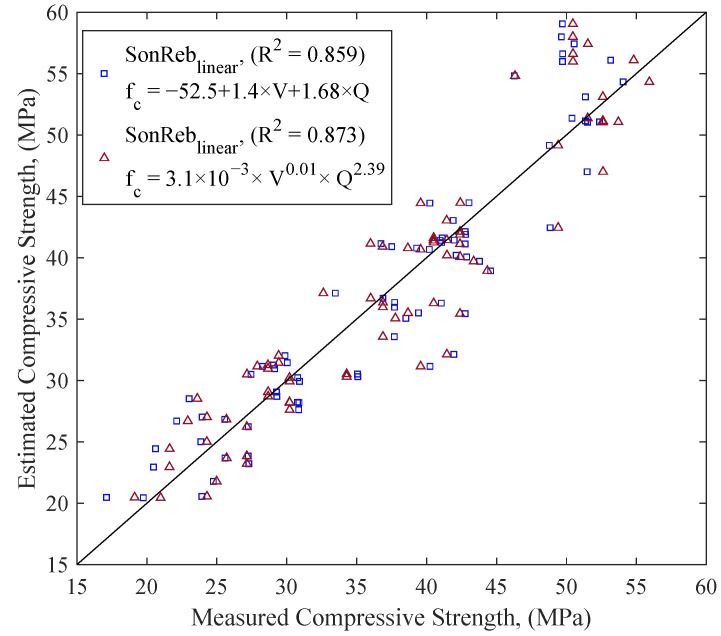
Comparison between measured compressive strength (f’_c_) and estimated strength (f’_cest_) using SonReb. Multivariate linear model, SonReb_linear_ (x), power model, SonReb_power_ (o).

**Figure 9:**
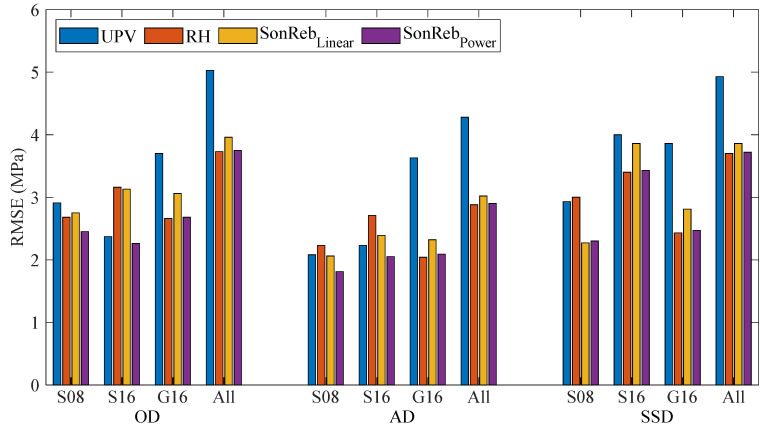
RMSE values of compressive strength estimation models using UPV, RH, and combined SonReb (_linear_ and _power_) methods. Results are shown for three saturation levels (OD, AD, SSD) and four aggregate groupings (S08, S16, G16, All), resulting in 12 datasets evaluated with four regression types each.

The combined use of UPV and RH (SonReb) was evaluated using multivariate regression models. The models used two types of mathematical forms: the multiple linear regression equation, SonReb_linear_, (Equation (4)), and the multiple power law model, SonReb_Power_, (Equation (5)). Multivariate regression results are provided as a comparison between the measured compressive strength ($f_{c}^{\prime }$) and the estimated compressive strengths ($f_{cest}^{\prime }$) (Figure 8). RMSE values of compressive strength estimation models were compared in Figure 9. In the figure, SonReb_linear_ is the multivariate regression model constructed following Equation (4), and SonReb_Power_ follows Equation (5). Figure 9 shows that SonReb_Power_ models performed better than SonReb_linear_ for most batches and saturation levels.

(4) $$f_{cest}^{\prime }=a+b_{1}\times \left(X_{1}\right)+b_{2}\times \left(X_{2}\right)$$

(5) $$f_{cest}^{\prime }=b\times {\left(X_{1}\right)}^{c_{1}}\times {\left(X_{2}\right)}^{c_{2}}$$

### 3.2. Correlation of Chloride Migration Coefficients with ER

**Figure 10:**
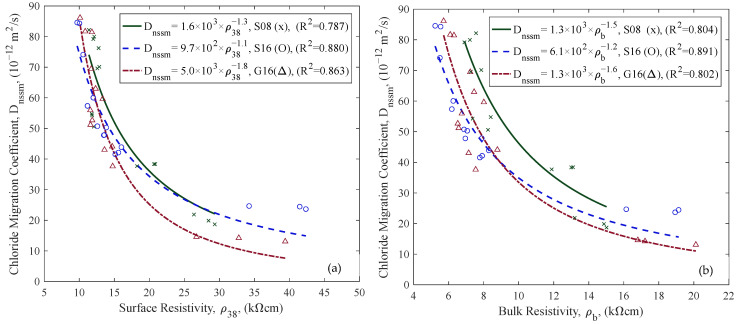
Relationships between D_nssm_ and (**a**) $\rho_{38}$ and (**b**) $\rho_{b}$ for different aggregate groups (green = S08, blue = S16, red = G16).

**Figure 11:**
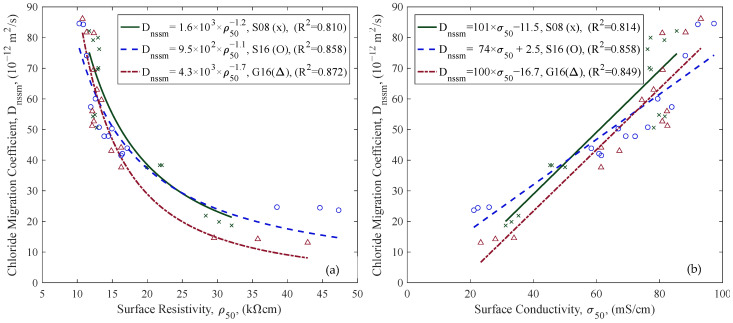
Relationships between D_nssm_ and (**a**) $\rho_{50}$ and (**b**) $\sigma_{50}$ for different aggregate groups (green = S08, blue = S16, red = G16).

The relationships between D_nssm_ and various resistivity measurements ($\rho_{38}$, $\rho_{50}$, $\rho_{b}$) in the SSD state for different aggregate compositions are shown in Figure 10 and Figure 11a. Power law regression models were fitted to each aggregate group (S08, S16, G16), showing strong correlations in all cases (R^2^ values ranging from 0.787 to 0.891). Conductivity, the reciprocal of resistivity (Equation (6)), was also correlated with D_nssm_ (Figure 11b). While the relationship between D_nssm_ and electrical resistivity is non-linear, it appears approximately linear with conductivity. Given the comparable correlation strengths, both parameters may be used for predictive purposes, as further discussed in the discussions.

(6) $$\rho =\frac{1}{\sigma }$$

ρ is the electrical resistivity,
*σ* is the electrical conductivity.

**Figure 12:**
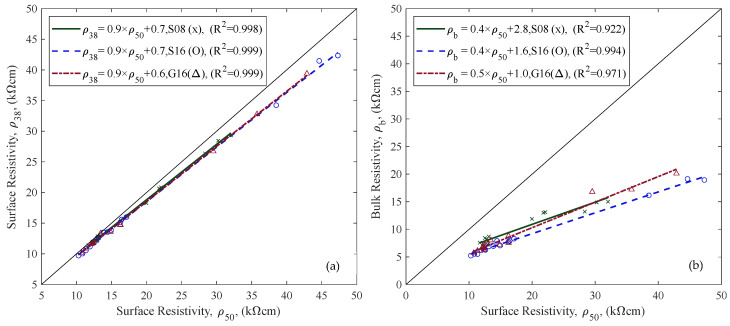
Comparison of ER measurements in the SSD condition: (**a**) $\rho_{38}$ with $\rho_{50}$, (**b**) $\rho_{bulk}$ vs. $\rho_{50}$.

**Figure 13:**
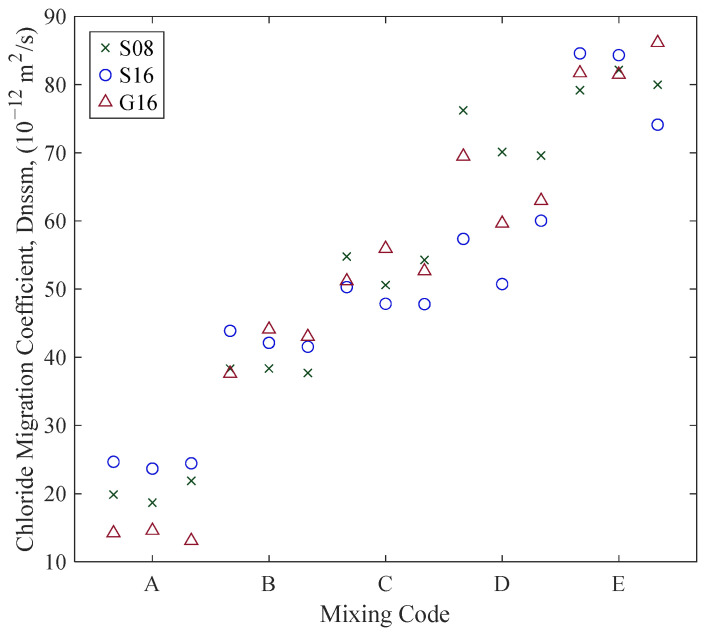
Chloride migration coefficients of concrete classes.

Different ER measurements ($\rho_{38}$, $\rho_{50}$, and $\rho_{b}$) are compared in Figure 12 to highlight differences in values obtained from surface and bulk configurations, as well as between probe spacings (38 mm and 50 mm). The correlation between $\rho_{38}$ and $\rho_{50}$ was very strong across all aggregate types, with R^2^ values exceeding 0.998, indicating a consistent proportional relationship. The correlations between $\rho_{b}$ and $\rho_{50}$ were slightly more variable but remained strong. These results confirm that all three configurations provide closely related values under SSD conditions. Additionally, measurements with larger electrode spacing produced higher resistivity values, and surface resistivity values were generally greater than bulk resistivity values. Lastly, D_nssm_ results of different concrete mix groups are provided in Figure 13, revealing chloride resistance differences across samples with varying w/c ratios and aggregate compositions (see Table 1).

## 4. Discussion

Recent studies emphasize the importance of test or prediction error, typically obtained through cross-validation or separate test sets, for non-destructive strength estimation of concrete [43,44,45]. However, the dataset in this study was not split due to its limited size, which would reduce the robustness of model fitting and subgroup comparisons. The primary aim was to investigate the influence of concrete parameters and develop comparative relationships under controlled conditions rather than to predict in situ performance. Nevertheless, field applications should rely on models validated with independent test data to ensure estimation reliability.

### 4.1. Effect of Aggregate Size and Type

Three aggregate compositions (S08, S16, and G16) with varying aggregate types and sizes were used to evaluate the effect of aggregate size and type. Results in the AD state indicate that aggregate characteristics significantly affect both UPV and RH measurements, with UPV showing greater sensitivity (cf. Figure 6), likely due to wave refraction and travel time distortion caused by internal heterogeneity [46,47]. Concrete specimens with sea stone (S16) consistently showed higher UPV and RH values than those with crushed granite (G16). These effects emphasize the importance of including aggregate information in reuse documentation. Knowing aggregate type and size improved the estimation accuracy (cf. Figure 9), aligning well with existing literature [31,48,49,50]. Therefore, detailing aggregate composition can contribute to the reliability of the reuse documentation.

Concrete samples with granite (G16) usually have lower resistivity values than those with sea stone aggregates (S08 and S16, cf. Figure 10 and Figure 11). These findings align with previous research [23,51], indicating that aggregate properties must be carefully considered in the ER-based durability documentation of concrete for reuse.

### 4.2. Effect of Saturation Level

The effect of saturation level on UPV and RH was investigated with three different saturation states: oven-dry (OD), air-dry (AD), and saturated surface dry (SSD) (Figure 7). The results indicate that saturation significantly influenced both UPV and RH outcomes, but in opposite directions. Consistent with earlier studies [52,53], UPV values increased with higher saturation levels, reflecting faster transmission due to increased density and pore saturation. Conversely, RH values decreased with increased saturation because moisture softens the concrete surface, reducing rebound values, consistent with previous literature findings [54].

These observed variations suggest the necessity of carefully evaluating saturation levels when documenting concrete properties for reuse. The opposite effects of saturation on UPV and RH indicate the potential benefits of combining the methods (SonReb), as this combination could mitigate uncertainties introduced by varying saturation conditions common in in situ assessments.

Additionally, ER measurements were conducted at the three different saturation levels. Stable ER measurements were only achievable in SSD level, whereas measurements in AD and OD states were unstable. This finding aligns with the literature, as ER measurements on samples with low saturation levels were found inappropriate [23,55]. The likely cause of instability is the high contact resistance between the electrodes and the dry concrete surface, which may not be adequately overcome by the limited voltage output (maximum 38 V) of the test device. Nevertheless, this limitation is a concern for in situ ER measurements where the saturation level varies. Consequently, evaluating durability-related properties based on field ER measurements requires careful evaluation of the saturation level of the concrete elements.

### 4.3. Combined (SonReb) or Single Methods

The combined use of UPV and RH (SonReb method) was compared with each method used individually (Figure 9). In our study, however, the SonReb method was the only method that consistently provided low RMSE values. The RH, in some cases, offered minor improvements in RMSE value, but it did not consistently provide low RMSE values across all regression analyses. This observation aligns with previous research indicating mixed results; some studies suggest combined methods offer enhanced accuracy [28,29,56,57,58], while others indicate limited or no significant benefit [59]. The effectiveness of the combined method in terms of estimation accuracy thus remains debated within the literature. When one method dominates in predictive accuracy, the improvement gained through combination may be limited, as the regression model tends to weigh the more predictive variable more heavily than the weaker one [60].

This limitation was evident in this study, as RH models provided consistently better prediction accuracy compared to UPV models across diverse aggregate compositions. UPV measurements are significantly influenced by aggregate type and size, limiting the accuracy improvement potential of the combined SonReb approach. Despite the relatively minor improvements in prediction accuracy, the combined method still offers substantial benefits beyond simple accuracy metrics.

One considerable advantage of combining UPV and RH is their opposing sensitivity to concrete moisture levels. Documentation of concrete elements for reuse mostly requires in situ testing, where the saturation level can vary, unlike laboratory testing, where concrete properties may be controlled. The combined approach can help neutralize these moisture-related measurement variations. This synergistic effect has been supported by previous studies [31,60,61] and offers clear benefits for practical reuse scenarios.

Reliability and consistency of results are crucial in the context of concrete reuse documentation, where a comprehensive evaluation is required. Relying on a single NDT method may be sufficient under limited time or resources; however, it creates risks due to limitations of the individual method and potential misinterpretation of results. Instead, the combined use can help reduce uncertainty and improve reliability, especially in cases with unknown aggregate size and type, and varying saturation levels (cf. Figure 9). Furthermore, the SonReb approach provides additional benefits, including the capacity to detect outliers more effectively by cross-checking results between methods. Even if one of the measurements is an outlier, the strength estimation of concrete can be evaluated through the second method.

### 4.4. Electrical Resistivity and In Situ Measurement

ER measurements were performed using three approaches: surface resistivity with 38 mm and 50 mm probe spacings, and bulk resistivity. Probe spacing of surface resistivity measurements influences the depth of the current flow: wider spacing generally leads to deeper, more homogeneous resistivity measurements [23]. However, wider probe spacing may increase the likelihood of interference from embedded reinforcement and geometrical effects of the sample size/shape [62]. The results indicated that a larger probe spacing showed higher resistivity values, aligning with findings in the literature [30,63]. A strong correlation was observed between surface resistivities with different probe spacing (Figure 12a).

Bulk and surface resistivity measurements also showed a strong correlation (Figure 12b), which agrees with the literature [63,64]. However, their field applicability differs significantly. Surface resistivity is more suitable for in situ applications due to its simpler and faster measurement process than bulk resistivity [65]. Therefore, surface resistivity has a higher potential for NDT reuse documentation than bulk resistivity.

Accurate documentation of concrete durability, specifically resistance to chloride ingress, is critical for structural reuse. Although ER is effective under controlled laboratory conditions, its effectiveness under field conditions may be compromised due to varying moisture levels. As ER measurements require high moisture levels for stability, field applications often face limitations.

On the other hand, indoor concrete elements have a lower risk of corrosion due to their limited exposure to moisture, absence of de-icing salts, and more stable temperature conditions [66,67]. Consequently, focusing initially on indoor concrete elements, which typically experience stable environmental conditions, could be an effective starting strategy for implementing reuse while comprehensive durability documentation methods are refined.

### 4.5. Scaling the Findings to Use in Full Scale

The main objective of this research was to support the NDT documentation of structural concrete elements for reuse to enable safe and scalable reuse of the structural elements. Valuable insights were obtained on the NDT methods, UPV, RH, and ER. Effects of concrete ingredients, such as aggregate type and size; and environmental conditions, such as saturation level, on relationships of NDT measurements with target properties were thoroughly investigated.

Concrete ingredients are often unknown in the field, and NDT measurements must be conducted without this knowledge. The findings showed that if the aggregate type and size are unknown, the estimation error metric, RMSE, rises consistently (Figure 9). Higher estimation error increases uncertainty, lowering the reuse value. Therefore, knowing the concrete ingredients can help to improve the reuse potential.

Nonetheless, the study indicates that the effects of aggregate type and size (Figure 6) and saturation level (Figure 7) were mainly limited to shifting relationships without changing the shape of the curve notably. Therefore, utilizing relationships from an NDT database calibrated with field results can improve the reliability of in situ strength assessment. Field results with a few core samples can be used to find the required shifting value, thus reducing the uncertainty caused by not knowing the ingredients and saturation.

In practice, comprehensive in situ concrete assessments are often constrained by limitations in core sampling. Reuse projects have even more limitations on core-sampling rate and location. Reuse assessments of concrete elements in the donor building should be conducted well in advance so that the receiver projects can be designed according to available material. Donor buildings, such as offices or residential buildings, might be assessed while inhabited. The assessment should be conducted with very few core samples to maintain the intended use of the building.

Moreover, core-sampling locations can be constrained by building owners or occupants of the inhabited buildings due to disruption from core drilling. This limitation might cause a reduction in the coverage of the whole measurement range suggested by standards such as EN 13791:2019 [68], leading to extrapolation and inaccuracy in estimations. Therefore, calibrating relationships from an NDT database with limited core sampling offers a practical solution to enhance reliability and accuracy for in situ reuse assessments. This approach can support reliable and practical reuse documentation. Thus, it can contribute to enabling safe and scalable reuse.

In addition, the reuse documentation can be enhanced by implementing systems for recording and storing detailed documentation of construction materials in new buildings. Such documentation would provide a valuable resource for future assessments when these buildings are eventually deconstructed, and the structural elements are considered for reuse.

## 5. Conclusions

Reliable and practical in situ documentation will play a crucial role in reuse projects by enabling the assessment of structural elements during their intended use. Non-destructive testing (NDT) can reduce the need for extensive and disruptive core sampling of traditional assessment approaches. Conducting NDT documentation within the donor building helps maintain its structural integrity and intended use while providing the necessary data on time for reuse project planning. This study addressed NDT documentation of concrete to enable the safe and scalable reuse of structural elements, with a strong emphasis on adapting experimental results into reliable and practical procedures for in situ reuse assessment within donor structures.

NDT methods such as ultrasonic pulse velocity (UPV), rebound hammer (RH), and electrical resistivity (ER) were investigated for in situ documentation of structural concrete elements intended for reuse. The study evaluated various parameters relevant to reuse projects, including the effects of saturation level and aggregate type and size on the relationships between NDT results and target properties. The study was conducted on concrete samples with a wide range of water–cement (*w*/*c*) ratios and three aggregate compositions. Compressive strength and chloride migration coefficient results were the target properties obtained through destructive tests. The results showed strong correlations between NDT measurements and the target properties. UPV and RH methods exhibited reliable estimates of compressive strength, while ER was effective in estimating the chloride migration coefficient, but only at high saturation levels.

Effects of saturation level and aggregate type and size on the NDT results highlight the importance of understanding the properties of the concrete being tested. Knowledge of these parameters improves the documentation for reuse as it decreases the uncertainty of NDT estimation. However, such information is often unavailable in practice.

In such cases, calibrating NDT database relationships using a limited number of core samples from the structure can enhance the reliability of in situ assessments. The findings of this study showed that variations in concrete ingredients and saturation levels primarily cause a shift in the relationships without significantly altering their overall shape. Therefore, even a small number of core samples can be used to determine required shifting, minimizing the impact of unknowns and improving the reliability of the evaluation.

Surface resistivity measurement is preferred for in situ NDT documentation due to its feasibility in field applications and strong correlation with D_nssm_. However, in situ resistivity measurements are less reliable than laboratory tests due to the strong effect of saturation on measurements. Therefore, focusing on reusing indoor concrete elements can be a practical strategy while comprehensive durability assessments are being developed.

The combined use of UPV and RH methods, also called SonReb, for estimating compressive strength showed limited improvement in regression metrics. However, improving estimation accuracy is not the only benefit of the combined use. Combined use can neutralize the influence of factors affecting NDT measurements in opposite directions, such as humidity. It can also be used for outlier analysis. Therefore, the combined use of NDT methods for reuse documentation is suggested.

## Figures and Tables

**Table 1:** Concrete mixture details.

Batch No.	Mixing Code	Concrete Constituent (kg/m^3^)								
		Cement	Water	*w*/*c*	Sand 0–4	Sea Stone 4–8	Sea Stone 8–16	Granite 4–8	Granite 8–16	SP *
**1**	S16A	363	163	0.45	850	380	600	-	-	3.1
**2**	S16B	310	183	0.59	850	380	600	-	-	2.2
**3**	S16C	287	187	0.65	850	380	600	-	-	1.6
**4**	S16D	265	195	0.74	850	380	600	-	-	0.6
**5**	S16E	241	202	0.84	850	380	600	-	-	0.0
**6**	S08A	363	163	0.45	730	1100	-	-	-	3.4
**7**	S08B	310	183	0.59	730	1100	-	-	-	2.2
**8**	S08C	287	187	0.65	730	1100	-	-	-	1.6
**9**	S08D	265	195	0.74	730	1100	-	-	-	1.3
**10**	S08E	241	202	0.84	730	1100	-	-	-	0.9
**11**	G16A	413	185	0.45	810	-	-	380	560	3.4
**12**	G16B	349	206	0.59	810	-	-	380	560	2.2
**13**	G16C	327	213	0.65	810	-	-	380	560	1.6
**14**	G16D	301	221	0.74	810	-	-	380	560	0.9
**15**	G16E	274	230	0.84	810	-	-	380	560	0.0

* Superplasticizer, mL/m^3^.

**Table 2:** Descriptive statistics of experimental results.

Test	Parameter	Sample Size (*n*)	Min.	Mean	Median	Max.	Std Dev.
Ultrasonic velocity (UPV)	V_OD_ (km/s)	75	3.582	3.908	3.912	4.296	0.192
	V_AD_ (km/s)	75	3.976	4.271	4.294	4.580	0.162
	V_SSD_ (km/s)	75	4.200	4.474	4.490	4.703	0.148
Rebound hammer (RH)	Q_OD_ (-)	75	43.0	52.0	52.5	61.0	5.2
	Q_AD_ (-)	75	38.0	49.7	50.5	59.5	5.7
	Q_SSD_ (-)	75	31.0	43.6	45.0	55.0	6.5
Compressive Strength	f_c_ (MPa)	75	20.43	37.21	36.30	59.06	10.54
Electrical Resistivity (ER)	$\rho_{50}$ (kΩcm)	45	10.28	18.37	13.12	47.30	10.02
	$\rho_{38}$ (kΩcm)	45	9.70	17.16	12.85	42.34	8.97
	$\rho_{b}$ (kΩcm)	45	5.23	9.43	7.56	20.11	4.18
Chloride Migration	D_nssm_ (×10^−12^ m^2^/s)	45	13.09	51.49	50.74	86.14	21.69

## Data Availability

The dataset will be publicly available after publication.
